# Multi-omics analysis reveals regulators of the response to nitrogen limitation in *Yarrowia lipolytica*

**DOI:** 10.1186/s12864-016-2471-2

**Published:** 2016-02-25

**Authors:** Kyle R. Pomraning, Young-Mo Kim, Carrie D. Nicora, Rosalie K. Chu, Erin L. Bredeweg, Samuel O. Purvine, Dehong Hu, Thomas O. Metz, Scott E. Baker

**Affiliations:** Earth and Biological Sciences Directorate, Pacific Northwest National Laboratory, Richland, WA USA

**Keywords:** *Yarrowia lipolytica*, Lipid, Proteome, Metabolome, Phosphorylation, Phosphoproteome, Nitrogen, Regulation, Beta-oxidation, Ribosome biogenesis, Translation

## Abstract

**Background:**

*Yarrowia lipolytica* is an oleaginous ascomycete yeast that stores lipids in response to limitation of nitrogen. While the enzymatic pathways responsible for neutral lipid accumulation in *Y. lipolytica* are well characterized, regulation of these pathways has received little attention. We therefore sought to characterize the response to nitrogen limitation at system-wide levels, including the proteome, phosphoproteome and metabolome, to better understand how this organism regulates and controls lipid metabolism and to identify targets that may be manipulated to improve lipid yield.

**Results:**

We found that ribosome structural genes are down-regulated under nitrogen limitation, during which nitrogen containing compounds (alanine, putrescine, spermidine and urea) are depleted and sugar alcohols and TCA cycle intermediates accumulate (citrate, fumarate and malate). We identified 1219 novel phosphorylation sites in *Y. lipolytica*, 133 of which change in their abundance during nitrogen limitation. Regulatory proteins, including kinases and DNA binding proteins, are particularly enriched for phosphorylation. Within lipid synthesis pathways, we found that ATP-citrate lyase, acetyl-CoA carboxylase and lecithin cholesterol acyl transferase are phosphorylated during nitrogen limitation while many of the proteins involved in β-oxidation are down-regulated, suggesting that storage lipid accumulation may be regulated by phosphorylation of key enzymes. Further, we identified short DNA elements that associate specific transcription factor families with up- and down-regulated genes.

**Conclusions:**

Integration of metabolome, proteome and phosphoproteome data identifies lipid accumulation in response to nitrogen limitation as a two-fold result of increased production of acetyl-CoA from excess citrate and decreased capacity for β-oxidation.

**Electronic supplementary material:**

The online version of this article (doi:10.1186/s12864-016-2471-2) contains supplementary material, which is available to authorized users.

## Background

The yeast *Yarrowia lipolytica* has the ability to accumulate a large fraction of its mass as neutral lipids, mainly in the form of triglycerides [1]. This, along with its genetic tractability, has made it an attractive model for the production of high value lipids and biofuel precursors [2, 3]. As in other fungi, lipid accumulation in *Y. lipolytica* is heavily dependent on environmental conditions, particularly the relative amounts and types of carbon and nitrogen sources available [1, 4–6].

The response to nitrogen quality and quantity has been well characterized in *Saccharomyces cerevisiae* [7–13] and is primarily regulated by nitrogen catabolite repression, which operates in yeast and filamentous fungi [14] to allow preferential utilization of preferred nitrogen sources. When high quality nitrogen sources are available, the GATA type transcription factors Gln3p and Gat1p become phosphorylated and remain bound to Ure2p in the cytosol, whereas they localize to the nucleus and activate nitrogen utilization genes in poor nitrogen conditions [15–18]. A second set of GATA type transcription factors, Gzf3p and Dal80p, act as repressors that compete for binding sites with and are regulated by Gln3p and Gat1p [19, 20]. It is unknown whether the pathways above operate similarly in *Y. lipolytica*. Interestingly, work comparing the evolutionary conservation of regulatory network motifs between *Y. lipolytica* and other yeasts, including *S. cerevisiae*, identified post-transcriptional regulation as the most conserved aspect of gene regulatory networks [21]. Comparison of the transcriptional regulators found that regulation of β-oxidation and peroxisome biogenesis by FarA and amino acid biosynthesis by Gcn4 are likely to be conserved between *Y. lipolytica* and filamentous fungi [22].

The purpose of this study was to reveal the regulatory changes of *Y. lipolytica* in response to nitrogen limitation. Though transcriptional responses to nitrogen limitation have been previously characterized in *Y. lipolytica* [4], the regulatory mechanisms driving these responses are not well understood. We analyzed the metabolome, proteome, and phosphoproteome in nitrogen replete and limited conditions to connect intracellular metabolite pools with the expression level and phosphorylation of proteins The analysis presented is focused on changes in the expression and phosphorylation state of regulatory proteins (kinases, phosphatases, and transcription factors) to generate hypotheses regarding which of these pathways are involved in regulation of the lipid accumulation response. To our knowledge, this is the first global study of protein phosphorylation in *Y. lipolytica* and as such, paves the way for future work on post-translational modification and protein engineering in this organism.

## Results

### Nitrogen limitation results in rapid increase in lipid droplet size

**Fig. 1:**
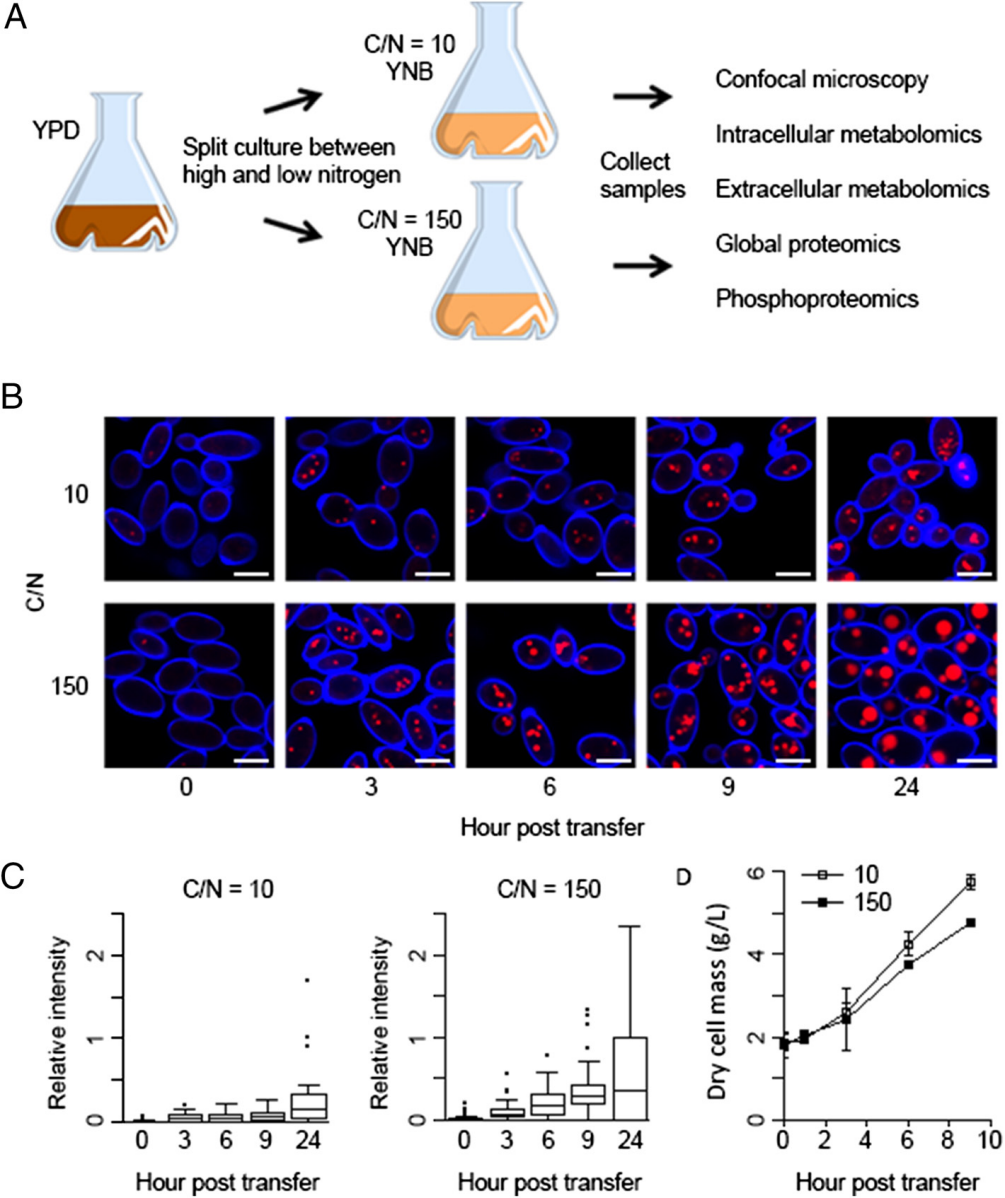
Experimental design for measuring the response to nitrogen limitation. **a** *Y. lipolytica* cultures grown in YPD are washed and split into YNB medium with a high (C/N = 10) or low (C/N = 150) concentration of ammonium sulfate to analyze the response to nitrogen limitation. After transfer cells were collected at regular time intervals and fixed for confocal microscopy or dried for mass measurement. We chose to collect samples at hour nine for metabolome and proteome experiments. **b** Fixed cells were stained with calcofluor-white (blue) to visualize the cell wall and nile-red (red) to image lipid droplets in response to nitrogen limitation. Bars indicate 5 μm. **c** Quantification of lipid droplet intensity. Box plot indicates the median, 10^th^, 25^th^, 75^th^ and 90^th^ percentiles. **d** Quantification of dry cell mass accumulation. Error bars represent the standard deviation of three replicates

It has been shown previously that limitation of nitrogen is an excellent and well conserved method to induce lipid accumulation in oleaginous fungi [5, 23, 24]. We have compared samples of the oleaginous yeast *Y. lipolytica* growing in either high (C/N = 10) or low (C/N = 150) nitrogen conditions to further our understanding of the metabolic events that lead from limitation of nitrogen to lipid accumulation. We grew *Y. lipolytica* in high (C/N = 10) or low (C/N = 150) nitrogen minimal medium followed by measurement of dry cell weight and microscopic examination (Fig. 1). Per cell lipid droplet staining intensity increased in high and low nitrogen conditions by three hours post transfer from rich medium (*p* < 0.001) and differed significantly by six hours post transfer (*p* < 0.001) (Fig. 1b and c). Cell mass accumulation was not significantly less in the nitrogen limited cells until hour nine (*p* < 0.001) (Fig. 1d). We chose to further explore the response to nitrogen limitation at hour nine, where lipid accumulation was clearly proceeding and cell mass accumulation was affected due to nitrogen limitation, by analyzing intracellular and extracellular metabolites as well as the global proteome and phosphoproteome.

### Metabolomic, proteomic and phosphoproteomic response to nitrogen limitation

**Fig. 2:**
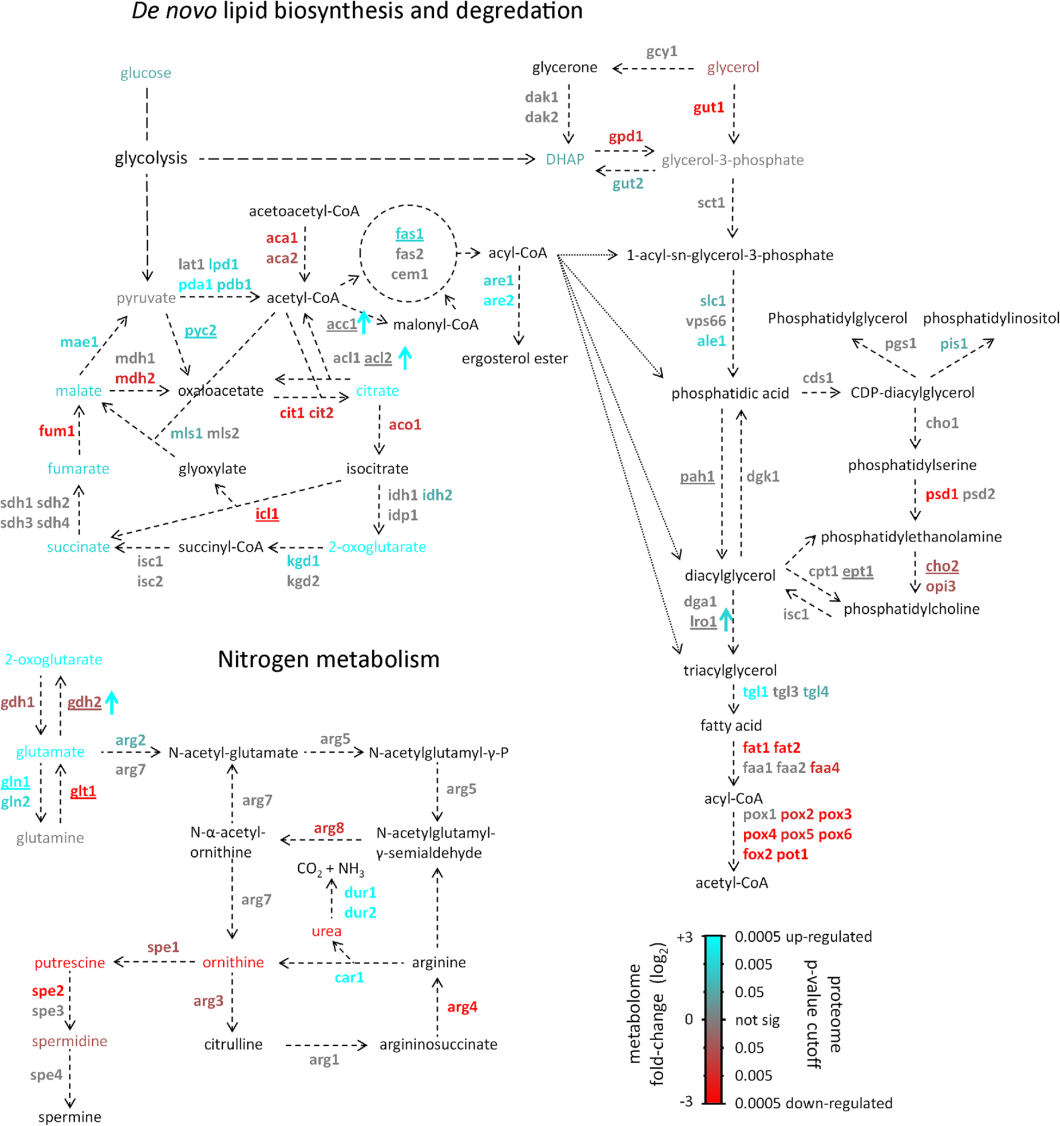
Response of the lipid and nitrogen metabolic networks to nitrogen limitation. Proteome, metabolome and phosphoproteome of *Y. lipolytica* grown in low (C/N = 150) versus high (C/N = 10) nitrogen conditions after nine hours. Nodes represent metabolites and edges represent genes. Colors of metabolites indicate log_2_ fold-change. Colors of gene names indicate significantly up and down regulated proteins identified by global proteomics; up-regulated indicates higher expression in the low (C/N = 150) nitrogen condition. Underlines indicate proteins with an identified phosphorylation site. Colored arrows indicate significant changes in phosphorylation level of a protein; up indicating more phosphorylation in the low (C/N = 150) nitrogen condition. Black text indicates metabolites and genes from which proteins were not identified. Metabolic pathways and gene names are based on homology to *S. cerevisiae* and *N. crassa*

We collected and analyzed extracellular and intracellular polar metabolites from *Y. lipolytica* 9 h after transfer to growth medium with either a high (C/N = 10) or low (C/N = 150) concentration of nitrogen in the form of ammonium sulfate, as well as after one hour of growth from the C/N = 150 condition. 146 intracellular metabolites were quantified, 93 of which were identified. 76 extracellular metabolites were quantified and 33 of these were identified. We also analyzed the proteome from *Y. lipolytica* after 9 h of growth in either high (C/N = 10) or low (C/N = 150) nitrogen conditions, and identified 59,578 unique peptides in at least one biological replicate. These peptides mapped to 4926 of the *Y. lipolytica* protein models in at least one replicate of the experiment and to 3567 of the models in all replicates, representing 55.3 % of the annotated coding sequences [25]. We identified 2101 unique quantifiable peptides after enrichment with immobilized metal affinity chromatography (IMAC). 1219 of those peptides had at least one phosphorylated serine, threonine or tyrosine residue and mapped to a total of 599 genes, paving the way for studies of protein phosphorylation in *Y. lipolytica*. The 882 peptides identified after IMAC enrichment but without a phosphorylation site are skewed toward the most abundant peptides identified in the phosphoproteomics analysis, suggesting they represent contamination from highly expressed proteins (Additional file 1) – these were not considered further. All quantified metabolites and peptides are tabulated in Additional file 2. We annotated and mapped *Y. lipolytica* genes to metabolic pathways relevant to nitrogen limitation and lipid accumulation and overlaid the metabolome, proteome and phosphoproteome data onto this map (Fig. 2).

### Nitrogen limitation results in nitrogenous metabolite depletion and intracellular carbon compound accumulation

**Fig. 3:**
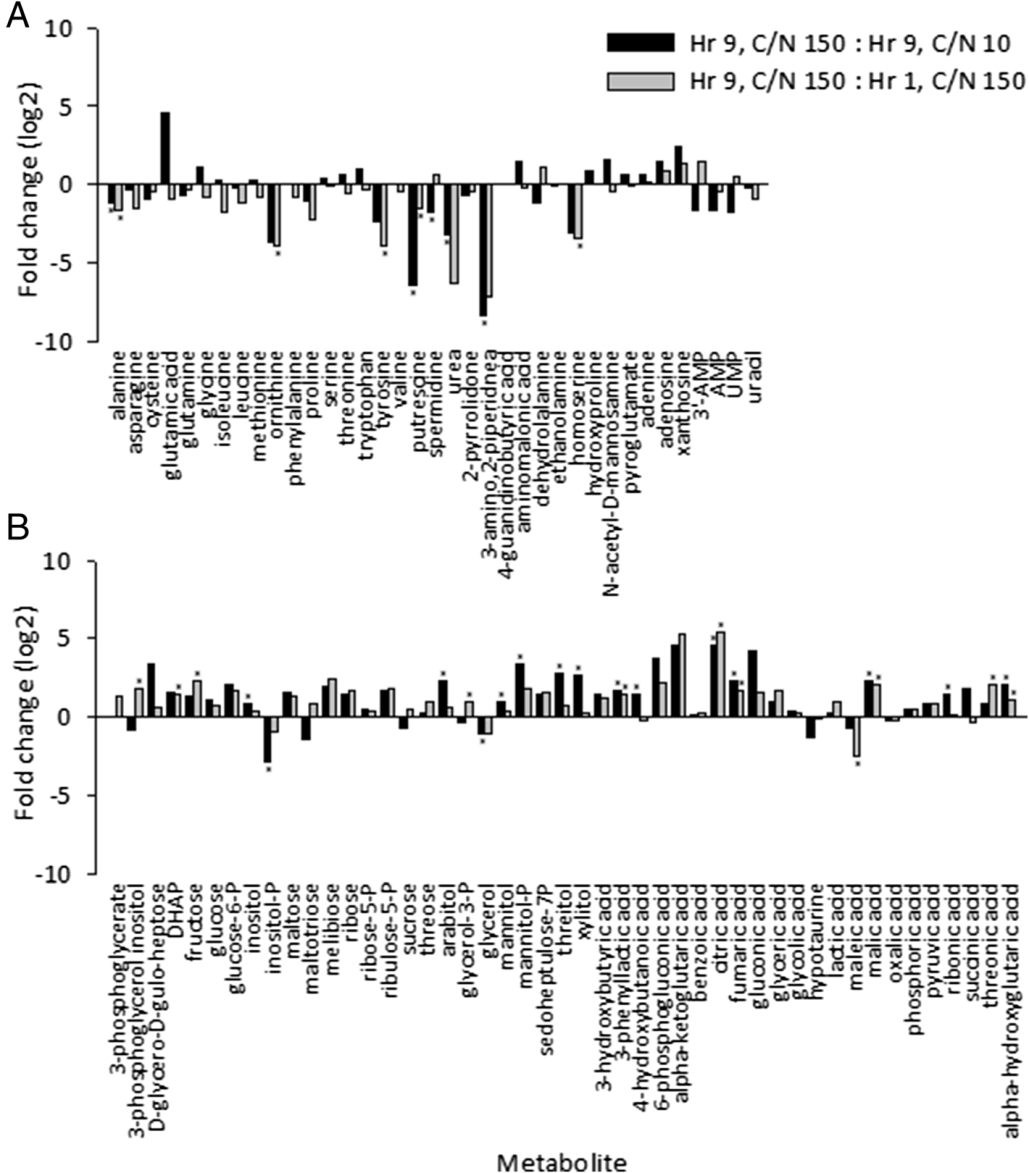
Intracellular metabolite pools in response to nitrogen limitation. Intracellular metabolite concentrations were measured and dry weight normalized after one and nine hours at C/N = 150 and nine hours at C/N = 10. Average fold change from three replicates each of (**a**) nitrogen containing metabolites and (**b**) non-nitrogen containing metabolites was calculated at nine hours for C/N = 150 vs. C/N = 10 (black bars) and for C/N = 150 at 9 vs. 1 h (grey bars). *significantly changing intracellular metabolites (*p* < 0.01). Arginine hydrolyzes to ornithine during chemical derivatization for GC-MS, thus ornithine represents a pooled value for intracellular ornithine and arginine

We found that nitrogen containing compounds tend to be depleted in cells grown in nitrogen limited medium (Hr 9, C/N 150 : Hr 9, C/N 10; Fig. 3a), while most non-nitrogen containing carbohydrates, alcohols and acids were present at higher intracellular levels in nitrogen limited medium (Hr 9, C/N 150 : Hr 9, C/N 10; Fig. 3b). Of the amino acids we measured, only alanine was significantly depleted in nitrogen limited cells, while the other significantly depleted nitrogen containing compounds between the C/N = 10 and C/N = 150 conditions at hour nine were 3-amino,2-piperidone, putrescine, spermidine and urea (Fig. 3a). We found arabitol, fructose, nigerose, threitol and mannitol at higher extracellular levels after 9 h than one hour in the C/N = 150 condition suggesting these metabolites are released by the cells (Additional file 2). Of these only threitol was at a significantly higher extracellular concentration after nine hours at C/N = 150 versus C/N = 10 suggesting threitol production is induced by nitrogen limitation. Intracellular threitol concentration is also significantly higher in the C/N =150 condition supporting this idea (Fig. 3b). Other significantly enriched metabolites found in the nitrogen limited cells include citrate, mannitol-P, xylitol, malate, fumarate, arabitol, α-hydroxyglutarate, phenyllactate, ribonate, γ-hydroxybutyric acid, and inositol (Hr 9, C/N 150 : Hr 9, C/N 10; Fig. 3).

### Proteomic response to nitrogen limitation

**Fig. 4:**
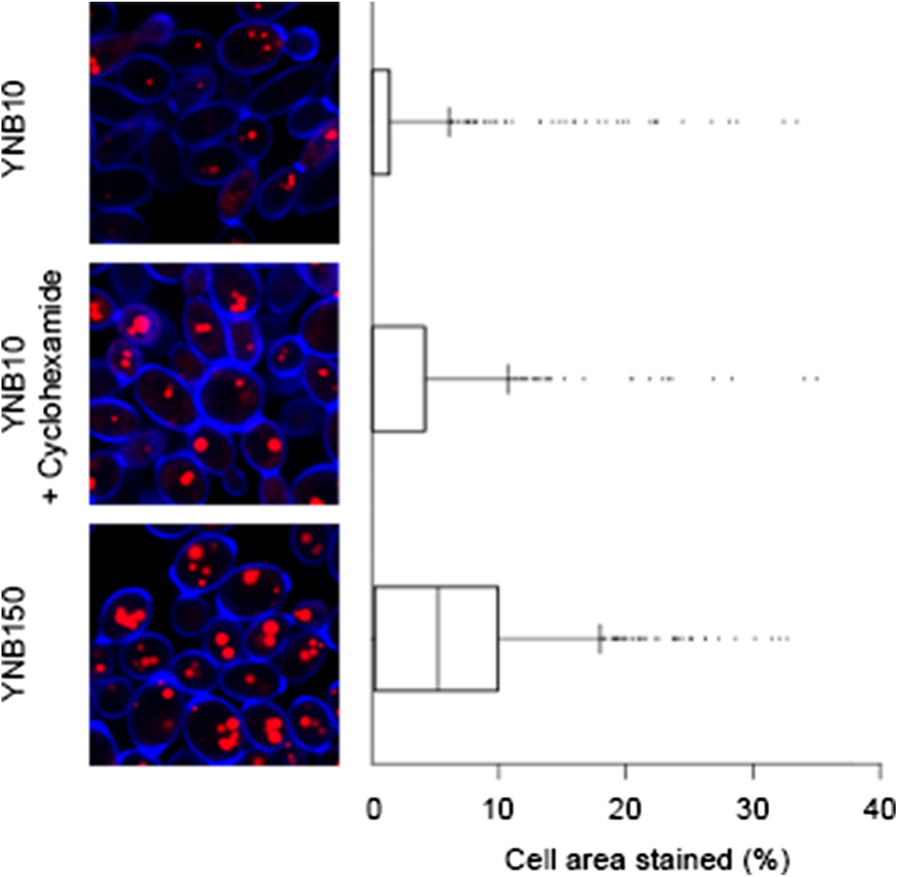
Inhibition of translation induces lipid droplet growth. *Y. lipolytica* cultures grown for nine hours were fixed, stained with calcofluor-white (blue) to visualize the cell wall and nile-red (red) to image lipid droplets. Cells were imaged in triplicate by confocal microscopy. Representative images are shown. The area of each cell covered by lipid droplets is calculated and statistically different in all three treatments (*p* < 0.05). Bars indicate 5 μm. Box plot indicates the median, 10^th^, 25^th^, 75^th^ and 90^th^ percentiles

**Fig. 5:**
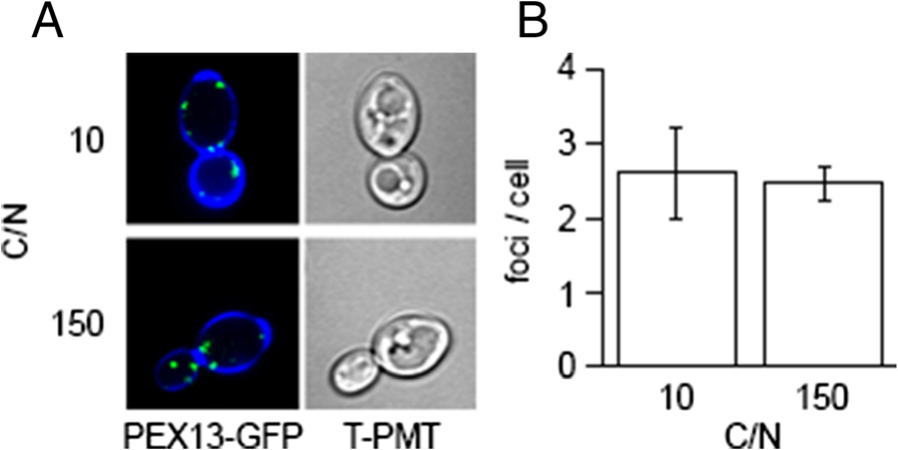
Number of peroxisomes does not change in response to nitrogen limitation. *Y. lipolytica* strain FEB64, harboring a green fluorescent protein tagged version of peroxisomal marker *pex13*, was grown in low (C/N = 150) and high (C/N = 10) nitrogen conditions as in Fig. 1. **a** At nine hours cells were collected, stained with calcofluor-white (blue), and imaged for Pex13-GFP (green). **b** The number of foci corresponding to peroxisomes per cell from two images from each of three replicates is not significantly different (*p* > 0.05)

We tested up- and down-regulated genes for gene ontology term enrichment and found that genes associated with proteolysis are up-regulated after nine hours of nitrogen limitation while amino acid metabolism, translation and ribosome biogenesis associated genes are down-regulated along with β-oxidation (Table 1) similar to what has been found in the oleaginous yeast *Rhodosporidium toruloides* [26]. We also found that of 115/132 proteins annotated as being a structural component of the ribosome are significantly down-regulated (*p* < 0.05) while none are significantly upregulated. We tested whether inhibiting translation would result in lipid accumulation in nitrogen replete conditions by addition of cycloheximide which blocks the elongation step in eukaryotic ribosomes [27]. We found that inhibition of ribosome function resulted in lipid droplet growth but to a lesser extent than nitrogen limitation (Fig. 4). This suggests that downregulation of the structural components of the ribosome, and presumably also a decreased capacity for translation, both play a part in lipid accumulation but are not entirely responsible. We found that proteins predicted to utilize acyl-CoA’s within the peroxisome for β-oxidation are downregulated, however, we did not see a change in the number of peroxisomes during lipid accumulation (Fig. 5) and so suggest it is only their function that is changing.

**Table 1:** Enriched biological process gene ontology terms. Analysis of up- and down-regulated genes (*p* < 0.01) for gene ontology term enrichment

Term	FDR	Representation
Up-regulated genes		
Proteolysis	5.70E-07	over
Translation	4.03E-06	under
Ribosome biogenesis	1.84E-05	under
Down-regulated genes		
Ribosome biogenesis	8.93E-21	over
L-serine metabolic process	1.11E-06	over
Glycine metabolic process	2.46E-05	over
tRNA aminoacylation for protein translation	3.29E-05	over
Lysine biosynthetic process	3.83E-05	over
Isoleucine biosynthetic process	1.05E-04	over
Regulation of translational initiation	1.87E-04	over
Valine biosynthetic process	1.25E-03	over
Leucine biosynthetic process	1.25E-03	over
Glyoxylate metabolic process	1.50E-03	over
Fatty acid β-oxidation	1.50E-03	over
Cysteine biosynthetic process	9.89E-03	over
Regulation of transcription, DNA-dependent	7.27E-04	under
proteolysis	1.34E-03	under

*FDR* false discovery rate after Fisher’s exact test

### Phosphoproteomic response to nitrogen limitation

We analyzed the 599 proteins with a phosphorylation site for gene ontology (GO) term enrichment to determine whether any particular types of proteins are prone to phosphorylation. Interestingly, this identified the process of protein phosphorylation and the functions of tyrosine and serine/threonine kinase activity as being very significantly enriched (*p* < 1E-10) suggesting the main function of phosphorylation in *Y. lipolytica* may be the regulation of kinases. We next compared the fold change values of the phosphorylated peptides with their counterparts in the global proteomics data to control for changes in protein expression and identify phosphorylation sites that change in their fractional abundance during nitrogen limitation. 53 peptides were identified as having decreased phosphorylation levels, while 80 exhibited increased phosphorylation (*p* < 0.05) during nitrogen limitation.

### Identification of regulatory pathways associated with nitrogen limitation

**Fig. 6:**
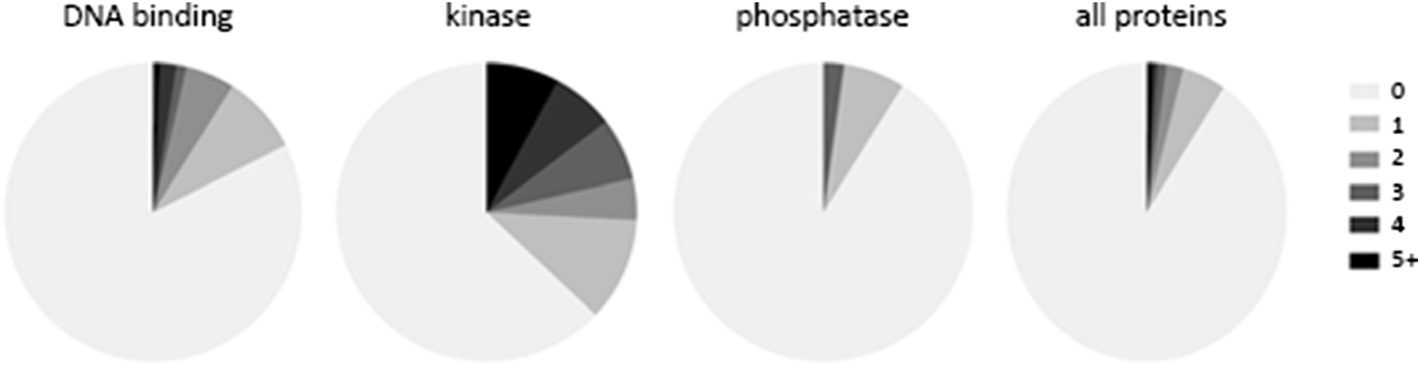
Regulatory proteins are enriched for phosphorylation. Genes were identified that are predicted to encode regulatory proteins (89 kinases, 45 phosphatases and 279 DNA binding proteins). The number of phosphorylation sites identified per protein in either of the conditions tested is quantified for each of these classes and the proteome as a whole

We are particularly interested in identifying the regulatory pathways affected by nitrogen limitation in *Y. lipolytica,* and to that end we predicted 89 kinases, 45 phosphatases and 279 DNA binding proteins present in *Y. lipolytica* using domain analysis and by comparison with homologs in other fungi. From those, 43 proteins (11 kinases, 2 phosphatase, 30 DNA binding proteins) were found to change significantly in their abundance (Table 2), while 23 peptides were found to change in their phosphorylation state (16 kinases, 7 DNA binding proteins) (Table 3) and which define a set of nitrogen responsive regulatory factors. We found that 37 % (33/89) of the predicted kinases and 18 % (49/279) of the predicted DNA binding proteins have at least one phosphorylation site, indicating that these potentially regulatory proteins are enriched for protein phosphorylation compared to the genome as a whole (9 %; 599/6448) but that phosphatases are not enriched for phosphorylation (9 %; 4/45). The number of phosphorylation sites identified for a given protein is also greater for kinases, and to a lesser extent, DNA binding proteins (Fig. 6).

**Table 2:** Regulatory proteins significantly changing in their abundance. Up- and down-regulated DNA binding proteins, kinases and phosphatases (*p* < 0.01). Best BlastP hits for *S. cerevisiae* and *N. crassa* are shown

		Change (Log_2_)	Std. Dev.	*S. cerevisiae*		*N. crassa*	
Gene	Blast2GO annotation			Gene	Symbol	Gene	Symbol
Up-regulated							
YALI0F16511g	dna-binding domain of mlu1-box binding protein mbp1	1.81	0.29	-	-	NCU06339	-
YALI0D20482g	nitrogen regulatory protein area	1.05	0.28	YER040W	GLN3	NCU09068	amr
YALI0E16577g	zinc-finger inhibitor of ho transcription	0.87	0.12	YKL185W	ASH1	-	-
YALI0F18788g	srf-type transcription factor	0.59	0.20	YBR182C	SMP1	NCU02558	-
YALI0C22682g	gata transcription factor	0.53	0.14	-	-	NCU15829	-
YALI0C11671g	nucleosome binding protein	0.52	0.10	YBR089C-A	NHP6B	NCU09995	-
YALI0E28897g	rna polymerase ii transcriptional coactivator	0.41	0.25	-	-	NCU04584	-
YALI0B20944g	c6 finger domain	0.38	0.14	-	-	NCU07675	-
YALI0A09020g	transcription factor	0.38	0.07	YNL257C	SIP3	NCU00495	-
YALI0D17996g	nhp10p	0.36	0.20	YDL002C	NHP10	NCU07568	-
YALI0C19151g	acetate regulatory dna binding protein	0.36	0.10	YMR280C	CAT8	NCU06656	acu-15
YALI0E08184g	cbf nf-y family transcription factor	0.34	0.09	YER159C	BUR6	NCU06405	pole-3
YALI0C22187g	membrane protein	0.34	0.10	YHR101C	BIG1	-	-
YALI0E18656g	c6 finger domain	0.33	0.18	-	-	-	-
YALI0E28721g	ssdna binding protein	0.26	0.14	-	-	-	-
YALI0F17468g	potential fungal transcription factor	0.22	0.09	-	-	NCU05051	col-23
YALI0A21241g	potential zinc finger protein	0.21	0.08	-	-	NCU02994	-
YALI0F11011g	rme1p	0.19	0.09	YGR044C	RME1	-	-
YALI0A10637g	fungal specific transcription factor domain-containing protein	0.19	0.06	-	-	NCU01478	-
YALI0F16852g	lim homeobox protein	0.17	0.04	-	-	NCU03593	kal-1
YALI0D14542g	camp-dependent protein kinase-	0.56	0.12	YHR205W	SCH9	NCU03200	stk-10
YALI0C04587g	protein kinase	0.53	0.22	YLL019C	KNS1	NCU00230	prk-4
YALI0D07150g	protein kinase	0.41	0.21	YKL116C	PRR1	NCU04143	stk-26
YALI0D25388g	serine threonine protein kinase	0.26	0.09	YGL180W	ATG1	NCU00188	apg-1
YALI0D16863g	casein kinase ii subunit alpha	0.23	0.06	YIL035C, YOR061W	CKA1, CKA2	NCU03124	cka
YALI0B04840g	serine threonine protein	0.17	0.08	YPL236C	ENV7	NCU07399	stk-9
YALI0F12617g	mitochondrially localized type 2c protein phosphatase	0.51	0.13	YHR076W	PTC7	NCU02749	-
Down-regulated							
YALI0F15169g	transcription factor	−0.52	0.17	YBR083W	TEC1	NCU02612	-
YALI0F05126g	phosphorus acquisition-controlling protein	−0.43	0.22	-	-	NCU09315	nuc-1
YALI0D05687g	mgmt family protein	−0.36	0.11	-	-	NCU11088	-
YALI0A17292g	air2p	−0.33	0.19	YDL175C	AIR2	NCU04617	-
YALI0E30789g	c2h2 finger domain	−0.33	0.18	-	-	NCU06503	-
YALI0E03432g	arc1p	−0.31	0.10	YGL105W	ARC1	NCU06307	-
YALI0D01573g	cell pattern formation-associated protein stua	−0.28	0.14	YMR016C	SOK2	NCU01414	asm-1
YALI0B12166g	multiprotein-bridging factor 1	−0.21	0.12	YOR298C-A	MBF1	NCU01422	mbf1
YALI0D07744g	YALI0D07744p	−0.20	0.12	-	-	-	-
YALI0F14267g	stromal membrane-associated protein	−0.15	0.09	YIL044C	AGE2	NCU03890	-
YALI0D19470g	mst3-like protein	−0.60	0.30	YDR523C	SPS1	NCU04096	prk-9
YALI0C00891g	serine threonine protein kinase	−0.52	0.15	YCR008W	SAT4	NCU06179	stk-5
YALI0C21758g	serine threonine protein kinase	−0.40	0.18	YPL141C, YOR233W	FRK1, KIN4	NCU00914	stk-16
YALI0C22770g	pkinase-domain-containing protein	−0.31	0.14	YNL298W, YOL113W	CLA4, SKM1	NCU00406	Vel
YALI0E06501g	agc akt protein kinase	−0.19	0.10	YKL126W, YMR104C	YPK1, YPK2	NCU07280	ypk1
YALI0B20438g	phosphoserine phosphatase	−0.34	0.10	YGR208W	SER2	NCU02004	ser-3

**Table 3:** Regulatory proteins significantly changing in their phosphorylation state. DNA binding proteins and kinases with a phosphorylation site detected in at least 2/3 biological replicates that significantly changes in abundance after controlling for global protein level (*p* < 0.05). Best BlastP hits for *S. cerevisiae* and *N. crassa* are shown

Changing phosphorylation sites		Blast2GO annotation	Change (Log_2_)	p-value	*S. cerevisiae*		*N. crassa*	
Gene	Phosphorylated peptide				Gene	Symbol	Gene	Symbol
YALI0D27258g	TSSIAQLSPTFSR	component of the 4 histone acetyltransferase complex	−2.25	0.00	YDR359C	EAF1	NCU07863	vid21
YALI0E16731g	STPIQTSQSPIQTR	YALI0E16731p	−1.11	0.03	-	-	-	-
YALI0D18678g	TRPASFSASSSASYLR	c2h2 transcription factor	−1.08	0.02	YML081W	TDA9	NCU09496	-
YALI0E05489g	GGAVPTFSDSPVRR	and fes cip4 domain protein	−0.68	0.00	YFL047W	RGD2	NCU09537	-
YALI0A19778g	VTGSPLVR	apses transcription	−0.63	0.00	YDL056W	MBP1	NCU07246	div-11
YALI0E18656g	ATTAFSPATAADFNYR	c6 finger domain	1.45	0.00	YDR520C	URC2	NCU01478	-
YALI0F11979g	TVGSPEYGSLLSR	rtg1p	2.08	0.00	YOL067C	RTG1	NCU02724	-
YALI0F00572g	DVSASPVFPK	serine threonine-protein kinase ste20	−1.16	0.00	YHL007C	STE20	NCU03894	stk-4
YALI0D07150g	TSILTTPPPAGR	protein kinase	−0.87	0.00	YKL116C	PRR1	NCU04143	stk-26
YALI0A00506g	ASTSLLSLTR	protein kinase	−0.85	0.03	YBL009W	ALK2	NCU00407	-
YALI0E06519g	APAQPLAPTQAVQSPPR	potential serine threonine-protein kinase hsl1	−0.85	0.00	YKL101W	HSL1	NCU09064	stk-53
YALI0E06519g	SYGSLLGSPVDAR	potential serine threonine-protein kinase hsl1	−0.84	0.00	YKL101W	HSL1	NCU09064	stk-53
YALI0A18590g	QSLITGSQPLPSPLR	serine protein kinase	−0.50	0.01	YMR216C	SKY1	NCU09202	mdk-2
YALI0C16665g	VYTYIQSR	protein kinase	−0.49	0.00	YJL141C	YAK1	NCU07872	prk-2
YALI0D07150g	YFNGNSPPMASISR	protein kinase	−0.45	0.03	YKL116C	PRR1	NCU04143	stk-26
YALI0D08822g	LVSDSQIDR	upstream serine threonine kinase for the snf1 complex	−0.41	0.01	YER129W	SAK1	NCU06177	camk-3
YALI0D20966g	ILVPGEPNVSYICSR	glycogen synthase kinase	0.25	0.02	YMR139W	RIM11	NCU04185	gsk-3
YALI0A10230g	SSITSTFSSSSNAIR	likely protein kinase	0.26	0.03	YNL183C	NPR1	NCU04335	stk-30
YALI0D08822g	SSTITNGILQR	upstream serine threonine kinase for the snf1 complex	0.29	0.00	YER129W	SAK1	NCU06177	camk-3
YALI0E27632g	SIDLLPNIR	calcium calmodulin-dependent protein	0.76	0.00	YOL016C	CMK2	NCU09123	camk-1
YALI0E26609g	LNATPPPLPEPAAVAR	casein kinase i	0.77	0.00	YNL154C	YCK2	NCU04005	ck-1b
YALI0A10230g	STSPVLNLAPHIQPGSGAEK	likely protein kinase	1.06	0.00	YNL183C	NPR1	NCU04335	stk-30
YALI0F27159g	GVDSGAVNFESLR	protein kinase	1.99	0.00	YJL057C	IKS1	NCU08177	stk-51

### Identification of DNA regulatory motifs associated with nitrogen limitation

**Fig. 7:**
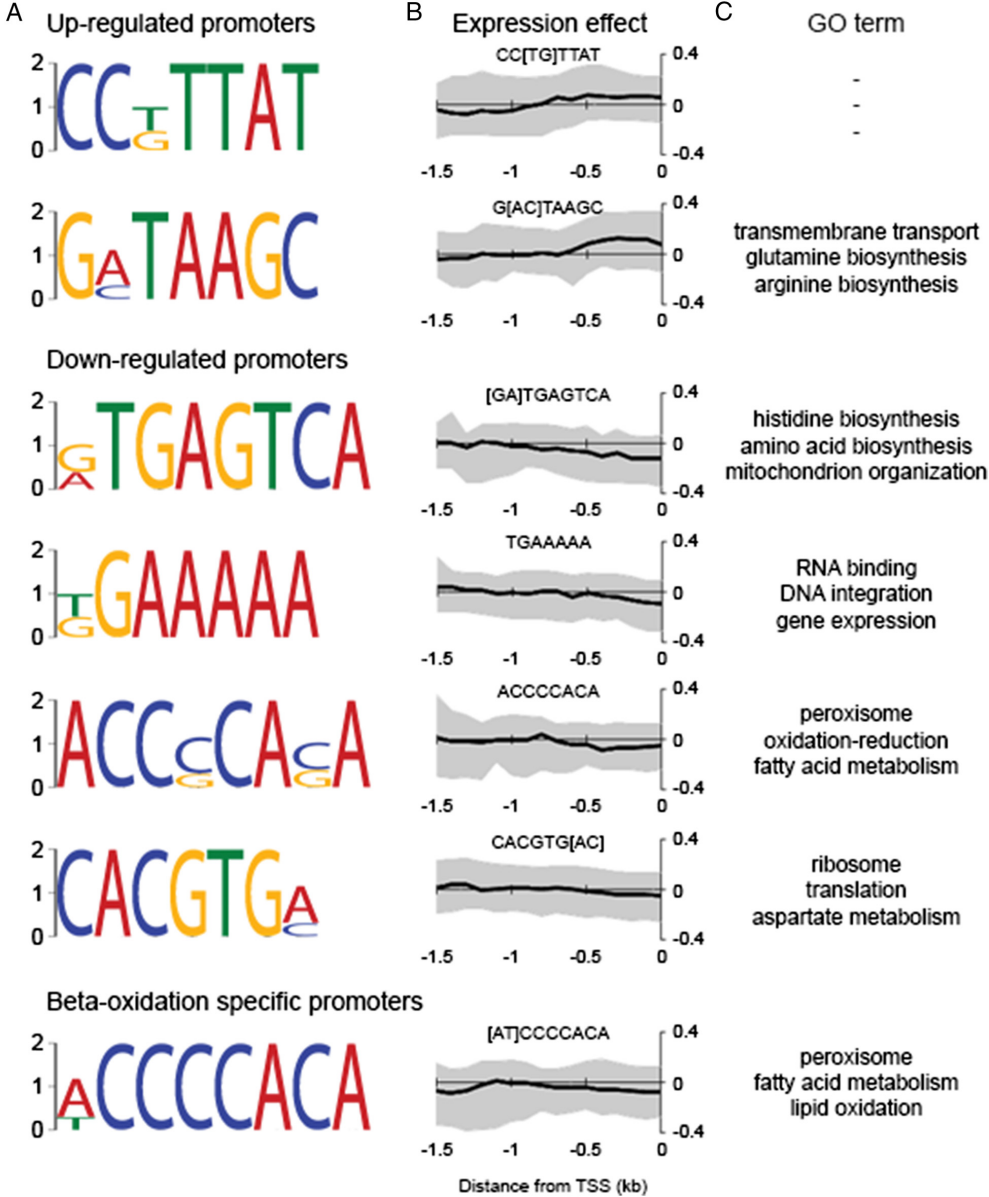
Promoter elements associated with nitrogen limitation responsive genes have distance dependent effects. **a** Promoter regions of genes that encode up- and down-regulated proteins as well as a subset of β-oxidation specific down-regulated proteins are enriched for specific DNA motifs. **b** For each *Y. lipolytica* gene the distance to the nearest occurrence of each motif five prime of the transcription start site is determined and binned in 0.1 kb windows based on this distance. The average protein fold change values for each bin up to 1.5 kb from the transcription start site for *Y. lipolytica* grown in low (C/N = 150) versus high (C/N = 10) nitrogen are plotted. The grey span indicates the interquartile range. **c** The most strongly enriched gene ontology (GO) terms (*p* < 0.005) for genes with the given DNA motif present within 0.5 kb 5’ of their transcription start site

We have found genes that are up- and down-regulated by limitation of nitrogen at the protein level. We were curious whether we could predict DNA elements driving these expression changes in the absence of RNA expression data. Thus, we analyzed the promoter elements of genes that were significantly up- and down-regulated for enrichment of short motifs. While this type of analysis is more appropriate for investigation of RNA expression data and in this context does not account for post-transcriptional regulation of expression, we identified two enriched motifs in the up-regulated genes and four enriched motifs in the down regulated genes as well as a motif specifically enriched in the promoters of β-oxidation genes (Fig. 7a), which are significantly down-regulated (Fig. 2). We validated the DNA motifs identified in the up- and down-regulated genes by comparing the effect of their position relative to the transcription start site (TSS) on expression level across all genes (Fig. 7b). We found that the presence of either a CC[TG]TTAT or G[AC]TAAGC site within roughly 0.5 kb of the TSS correlates with up-regulation, while a [GA]TGAGTCA, TGAAAAA, ACCCCACA or CACGTG[AC] site correlates with down-regulation. The [AT]CCCCACA site identified in the β-oxidation specific promoters is similar to the ACCCCACA site found in the down-regulated genes and is associated with down-regulation in both cases.

### Prediction of function of DNA motifs associated with nitrogen limitation

The function of the CC[TG]TTAT motif in the up-regulated genes is unclear. The second motif, G[AC]TAAGC is clearly a binding site for GATA family transcription factors. Of the 10 predicted GATA family transcription factors in *Y. lipolytica* three are up-regulated (YALI0C22682g, YALI0D20482g, YALI0E16577g) and three are down-regulated (YALI0E30547g, YALI0F14267g, YALI0F17886g) during nitrogen limitation. We hypothesize that one or more of these six proteins in combination regulate genes with a G[AC]TAAGC site in their promoter region. Included in these GATA transcription factors are three proteins with similarity to Gln3p in *Saccharomyces cerevisiae* [28] and AreA in *Aspergillus nidulans* [29] both of which regulate nitrogen catabolite repression in these organisms [30, 31].

The functions of the motifs in the down-regulated genes are also predictable. The [GA]TGAGTCA motif is similar to sites in *S. cerevisiae* that are regulated by nitrogen responsive transcription factors that regulate amino acid biosynthesis including Gcn4p, Arg81p, Arg80p and Cup9p [32–35]. We did not identify significant changes for homologs of these genes in *Y. lipolytica*. However, the ortholog of *gcn4* (YALI0E27742g) has a phosphorylation site at S219 that decreases during nitrogen limitation. We did not detect peptides specific to Gcn4 in our global proteomic data and were thus unable to calculate the fractional change for this phosphorylation site. However, in nitrogen replete conditions in *S. cerevisiae*, Gcn4p is rapidly turned over by a process dependent on phosphorylation of the proteins activation domain and subsequent degradation by the 26S proteosome [36]. Our results indicate a similar mechanism controlling amino acid metabolism is active in *Y. lipolytica*.

The [TG]GAAAAA motif resembles the ribosomal RNA processing element, bound by Stb3p in *S. cerevisiae* [37, 38]. The homolog in *Y. lipolytica* (YALI0C09232g) has a number of detected phosphopeptides, none of which change significantly. This is in contrast to studies in *S. cerevisiae* where nutrient limitation results in decreased phosphorylation of Stb3p by Sch9p as a mediator of ribosome biogenesis [39].

The ACCCCACA motif resembles that bound by the carbon catabolite repressor Mig1p (in *S. cerevisiae*) or CreA (in *A. nidulans*) [35, 40, 41]. We did not detect significant changes in the protein level of the Mig1 homolog in *Y. lipolytica* (YALI0E07942g). However, it may be differences in regulation rather than protein level that contribute to the nitrogen limitation response. Deletion of *mig1* in *Y. lipolytica* results in lower expression of β-oxidation genes at the RNA level and increased lipid accumulation [42] Together, these results suggest Mig1 is a direct regulator of β-oxidation genes in *Y. lipolytica*.

The CACGTG[AC] motif resembles the E-box motif originally described as an enhancer in mammals [43] and subsequently found to be bound by a number of basic helix-loop-helix (bHLH) transcription factors in yeast including Cbf1p, Tye7p, Hac1p, Rtg3p, Ino2p, Pho4p and Ino4p [34, 35, 44]. Of the nine predicted bHLH family transcription factors in *Y. lipolytica*, two are up-regulated (YALI0D15334g and YALI0F11979g), one is down-regulated (YALI0F05126g) and the homolog of Rtg1p (YALI0F11979g) is significantly phosphorylated during nitrogen limitation.

## Discussion

### Nitrogen limitation induced lipid accumulation

Biochemical investigations into the nature of oleaginous fungi has found that lipid accumulation occurs when environmental nitrogen is depleted in the presence of excess carbon, glucose in this case. When nitrogen is limited, the cells continue to utilize glucose but will no longer divide. Excess carbon flow into the tricarboxylic acid (TCA) cycle accumulates as citrate which is transported from the mitochondria to the cytosol and utilized for *de novo* production of fatty acids [45]. Both decreased capacity for amino acid biosynthesis for protein production and polyamine biosynthesis, which are essential for nuclear division [46, 47], may prevent cell division. However, the ultimate reason for decreased division may depend on initial environmental conditions that determine which intracellular nitrogenous metabolite becomes limiting first. After nine hours of nitrogen limitation, we found that all three *Y. lipolytica* cyclins are significantly down-regulated (Additional file 2), suggesting fewer cells are in G1, S or G2 phase. Examination of the TCA cycle in an oleaginous yeast such as *Y. lipolytica* would suggest that αlpha-ketoglutarate would accumulate when ammonium is not available for fixation to glutamate. We found this to be the case, along with accumulation of all other detected TCA cycle intermediates (Figs. 2 and 3), highlighting that carbon intermediate build-up occurs throughout the TCA cycle and is not specific to citrate [48]. Accumulation of citrate is thought to promote lipid accumulation through the production of acetyl-CoA via ATP-citrate lyase [49]. In support of this idea, we found that Citrate Synthase, which utilizes acetyl-CoA to make citrate from oxaloacetate, and Aconitase, which isomerizes citrate to isocitrate, are down-regulated, suggesting flux through these reactions is proceeding at a lower rate (Fig. 2).

We did not find a difference in expression of any of the enzymes involved in *de novo* lipid production from citrate. Rather, ATP-Citrate Lyase and Acetyl-CoA Carboxylase are phosphorylated (Fig. 1, Additional file 2), suggesting that if their activity is changing due to rapid accumulation of lipids, it is due to posttranslational modification of the enzymes. Overexpression of ATP-Citrate Lyase [50] and Acetyl-CoA Carboxylase [51, 52] have yielded positive results when applied to lipid accumulation in *Y. lipolytica*. However, if the activity or localization of these enzymes is regulated by phosphorylation, then it may be possible to achieve better results using strains where the phosphorylated residue(s) have been mutated to mimic constitutively phosphorylated or unphosphorylated enzymes. Work in mammals has identified phosphorylation of ATP-citrate lyase by insulin responsive pathways as a regulator of fatty acid biosynthesis in the liver [53, 54], and global analysis of protein phosphorylation has identified numerous sites on Acetyl-CoA Carboxylase that are subject to modification in *S. cerevisiae* [55], some of which have been mutated in recent attempts to enhance lipogenesis in *S. cerevisiae* [56, 57]. An additional general driver of lipid synthesis in oleaginous organisms is thought to be Malic Enzyme which oxidizes malate to pyruvate and CO_2_ and reduces NADP^+^ to NADPH - the production of NADPH as a reducing agent for fatty acid synthesis being the key aspect of this reaction [58]. In agreement, we found the gene for Malic Enzyme to be upregulated in *Y. lipolytica* during lipid accumulation (Fig. 2).

While the enzymes for synthesis of acyl-CoA from acetyl-CoA appear to be controlled by phosphorylation, the production of storage lipids is a different story. *De novo* production of triglycerides can proceed by repeated acylation of glycerol, which becomes the backbone. However, production of glycerol-3P via either phosphorylation of glycerol or reduction of DHAP is unlikely as both enzymes are down regulated during nitrogen limitation. Instead, the expression of Slc1 and Ale1, which make phosphatidic acid, and Are1 and Are2, which acylate sterols to steryl-esters, increases during nitrogen limitation, suggesting that most of the acyl-CoA produced is being transferred to sterols and 1-acyl-sn-glycerol-3P. This suggests that, for production of neutral lipids rather than sterol esters, it may be useful to engineer strains with lower expression of Are1/2.

### Nitrogen assimilation

Of particular interest is the effect of limitation of extracellular nitrogen on intracellular nitrogen carriers and intracellular sensing and signaling in response to depleted metabolites. Nitrogen assimilation occurs via either (1) NADP dependent Glutamate Dehydrogenase (Gdh1), which utilizes NADP^+^ to convert αlpha-ketoglutarate and NH_3_ to glutamate, or (2) Glutamine Synthetase (Gln1/2), which produces glutamine from glutamate and NH_3_ with energy from ATP. During nitrogen limitation we found both Gln1 and Gln2 to be strongly up-regulated, while Gdh1 was somewhat down-regulated suggesting route (2) is used for nitrogen assimilation during nitrogen starvation, as has been reported previously in other organisms [59–61]. Glutamate Synthase (Glt1) which drives the production of two molecules of glutamate from glutamine and αlpha-ketoglutarate, thus completing the cycle of formation of glutamate from αlpha-ketoglutarate and NH_3_, is down-regulated, suggesting the cells are limited for glutamine rather than glutamate and are free to make it using ATP when glucose is available in excess.

Of the amino acids we detected, only alanine is significantly depleted in nitrogen limited cells while the other significantly depleted nitrogen containing compounds we measured are 3-amino, 2-piperidone, putrescine, spermidine and urea. Urea production occurs via Arginase mediated degradation of arginine to ornithine and urea [62] or as a biproduct of purine catabolism [63]. The decrease in urea confirms that ammonium is not available in excess in the C/N = 150 condition as expected. The physiological relevance of 3-amino,2-piperidone in fungi is unclear; however, it is found at elevated levels in *Homo sapiens* with a defect in *SLC25A15,* which transports ornithine across the mitochondrial membrane [64, 65], suggesting it may be involved in urea cycling. The polyamines putrescine and spermidine are produced from ornithine via the activity of Ornithine Decarboxylase (*spe1*) and Spermidine Synthase (*spe3*) [66] and their production is regulated at the level of *spe1* in *Neurospora crassa* [67]. Putrescine and spermidine decrease during nitrogen limitation, as does intracellular ornithine to a less significant degree; these decreases occur concurrently with down-regulation of *spe1* and *spe2* (Figs. 2 and 3). This result is unexpected when compared to studies in *N. crassa* where polyamine starvation results in high *spe1* activity [68] but may reflect repression due to low ornithine availability during nitrogen limitation.

### Kinase signaling in response to nitrogen limitation

Categorically, we observed the highest number of phosphorylation sites on kinases. In total we identified 16 kinases that change significantly in their phosphorylation state and thus can begin to construct a network of phosphorylation dependent signaling cascades in *Y. lipolytica*. We compared the significantly changing phosphorylation sites with the wealth of phosphorylation data available for *S. cerevisiae* through Phophogrid [69] but found most (10/16) occur in regions of the proteins without significant homology between *S. cerevisiae* and *Y. lipolytica*. We can say little about the function of the sites we observed that change in their phosphorylation state in the absence of clear homology with other organisms. However, global studies on protein-protein interactions and functional studies using phosphosite mutants will aid our effort to construct a complete regulatory network for Yarrowia. Of the six phosphorylated sites that are in conserved regions between *S. cerevisiae* and *Y. lipolytica*, four have not been observed to be phosphorylated and two have been observed as phosphorylated on a homologous site in *S. cerevisiae*. These two phosphorylation sites are present in homologs of Rim11p (YALI0D20966g) and Yak1p (YALI0C16665g) and in both cases are required for, or enhance, the kinase activity of the enzyme. Phosphorylation of Tyr-181 in YALI0D20966g is a conserved site of modification that regulates the activity of the glycogen synthase kinase 3 enzymes Rim11p (at Tyr-199 in *S. cerevisiae*) and GSK-3B (at Try-216 in *H. sapiens*). In both yeast and humans phosphorylation is associated with an increase in the activity of the kinase [70, 71], suggesting that in *Y. lipolytica* this kinase is activated during nitrogen limitation. Phosphorylation of Tyr-583 of YALI0C16665g is homologous with Tyr-530 phosphorylation in *S. cerevisiae* Yak1p, which is autophosphorylated, resulting in kinase activation [72]. Dephosphorylation of YALI0C16665g suggests the activity of a Yak1-like pathway is decreased during nitrogen limitation.

### Regulatory response to nitrogen limitation

The effect of limiting nitrogen on the transcriptome has been previously characterized in *Y. lipolytica* and results in changes in transcripts related to nitrogen metabolism along with repression of protein production [4]. However, the regulatory factors driving these changes have not been previously investigated in *Y. lipolytica*. Our results show that the response to nitrogen limitation involves changes in the intracellular metabolite pools that affect the expression and activity of genes through specific transcription factor binding domains and kinase signaling. We identified short DNA elements that associate GATA family transcription factors with up-regulated genes and leucine zipper family transcription factors with down-regulated genes (Fig. 7). GATA family transcription factors primarily control amino acid biosynthesis in *S. cerevisiae* [12]. This function appears to be conserved in *Y. lipolytica* as genes with a G[AC]TAAGC motif within 500 base pairs 5’ of their transcription start site are enriched for amino acid biosynthetic processes (Fig. 7). Amino acid biosynthetic GO terms were also identified for upregulated genes with a [GA]TGAGTCA site in their promoter that is similar to that bound by Gcn4p [32, 73], which activates genes in response to amino acid starvation [33]. In the nitrogen limited cells, genes with the [GA]TGAGTCA motif tend to be down- rather than up-regulated suggesting the nitrogen replete cells represent conditions more akin to amino acid starvation or that this motif represents regulation by a novel pathway in *Y. lipolytica*.

A motif similar to that bound by the carbon catabolite repressor creA, ACCCCACA, is associated with genes that are down-regulated during nitrogen limitation. This is quite interesting as the sole carbon source for the experiment is glucose. During nitrogen limitation, the intracellular concentration of glucose and glucose-6P is higher which may explain greater repression of genes with the ACCCCACA motif in their promoter. Depictions of carbon catabolite repression in *S. cerevisiae* often emphasize repression of genes involved in utilization of alternative carbon sources [12]. In *Y. lipolytica* the alternative carbon source genes are those associated with fatty acid metabolism and peroxisomal β-oxidation which makes sense in light of the fact that *Yarrowia* is often isolated from lipid rich substrates [74–76] and whose metabolism is geared toward utilization of fats [77].

## Conclusion

In summary, we have used a variety of techniques to characterize the response to nitrogen limitation in the oleaginous yeast *Y. lipolytica*. In our growth conditions we observed complex changes in intra- and extracellular metabolite levels during batch culture and correlate these with microscopically observed cellular features and changes in protein expression and phosphorylation state. Substantial effort has been applied in the past few years to understanding the nature of and engineering oleaginous yeast [4, 6, 52, 78, 79], but to date only limited work has been done to characterize lipid accumulation from a regulatory perspective. Here we provide a comprehensive dataset describing nitrogen limitation induced lipid accumulation in *Y. lipolytica* that will enable more detailed experiments to understand the oleaginous nature of *Yarrowia* and provide genetic targets for future metabolic engineering efforts.

## Methods

### Yeast strains, cultivation and sample collection

Wild-type *Y. lipolytica* strain W29 (ATCC20460^™^) (American Type Tissue Culture; Manassas, VA) was used for metabolome and proteome experiments. Strains were maintained in YPD medium (1 % yeast extract, 1 % peptone, 2 % glucose) at 28 °C. Frozen stocks were maintained at −80 °C in 25 % glycerol. For experiments, *Y. lipolytica* was pregrown overnight in YPD broth in baffled 250 mL shake flasks at 28 °C and 200 rpm. The cells were pelleted and washed twice with 1.7 g/L yeast nitrogen base without amino acids and ammonium sulfate (BD; Franklin Lakes, NJ) and inoculated into 50 mL YNB10 (1.7 g/L yeast nitrogen base without amino acids and ammonium sulfate, 25 g/L glucose, 5.5 g/L ammonium sulfate) or YNB150 (1.7 g/L yeast nitrogen base without amino acids and ammonium sulfate, 25 g/L glucose, 0.366 g/L ammonium sulfate) to OD_600_ = 5 in baffled 250 mL shake flasks at 28 °C and 200 rpm in triplicate. The concentration of ammonium sulfate affects the pH of the medium. YNB10 is pH 5.4 while YNB150 is pH 4.9. Cycloheximide was added to 100 μg/mL when appropriate. For protein and intracellular metabolite measurements, 10 mL samples were collected by vacuum filtration on 0.45 μm nylon Whatman filters (GE Healthcare; Little Chalfont, UK) and washed with 5 mL 1.7 g/L yeast nitrogen base without amino acids and ammonium sulfate. The filters were transferred to Eppendorf tubes, weighed and then flash frozen in liquid nitrogen. For extracellular metabolites, cells were pelleted at 22,000 × g for 1 min and 1 mL of supernatant was collected and frozen in liquid nitrogen. For microscopy, 1 mL samples were collected and fixed in 4 % formaldehyde (Ted Pella; Redding, CA) for 30 min, washed 3 times with PBS (8 g/L NaCl, 2.56 g/L Na_2_HPO_4_ · 7H2O, 0.2 g/L KCL, 0.2 g/L KH_2_PO_4_) and then stored at 4 °C. For dry weight, 1.5 mL samples were washed once with H_2_0 and their weight measured after drying the cells in a speed vac.

### Strain construction

PCR used Q5 high fidelity DNA polymerase (New England Biolabs; Ipswich, MA). PCR products were purified using the GeneJET gel extraction and DNA cleanup micro kit (Thermo Scientific; Rockford, IL). Transformations were performed using a quick version of the lithium acetate transformation procedure [80]. Transformants were selected on YNB10 agar and verified by PCR and microscopy. The *pex13* ortholog (YALI0C05775g) was -GFP tagged using a split marker strategy. The 5’ and 3’ sequences flanking the end of *pex13* and the entire *leu2* gene were amplified from W29 gDNA with primers OEB172 (GGCTACGGAGGCATGAACT) and OEB173 (CCTCCGCCTCCGCCTCCGCCGCCTCCGCCATTAACGGGAACGGCACTG), OEB174 (TGCTATACGAAGTTATGGATCCGAGCTCGACACATACTGGTGCGACTGC) and OEB175 (TCGTTTTCGTCCTTCACCTC), and OEB170 (CGTAACCCTGATCGTACCTTGATGTCGACCCGTTGCTATCTCCACAC) and OEB171 (CGAGCTCGGATCCATAACTTCGTATAGCAGTGCAGTCGCCAGCTTAAA) respectively while *gfp* was amplified with primers OKP31 (GGCGGAGGCGGCGGAGGCGGAGGCGGAGG) and OEB169 (CGACATCAAGGTACGATCAGGGTTACGGATATCTTACTTGTAAAGCTC) from plasmid pYL4. These were then assembled into *pex13*-*gfp*:*leu2* split marker cassettes using overlapping PCRs and transformed into strain FKP355, derived from the Po1g [81] genetic background in which *ku70* was replaced with *hph* (Bredeweg EL, Pomraning KR, Dai Z Baker SE, in prep).

### Confocal microscopy

Fixed cells were stained in PBS with nile red and calcofluor-white and visualized without additional washing using a Zeiss LSM710 confocal laser-scanning microscope (Carl Zeiss MicroImaging GmbH, Munchen, Germany) and Plan-Apochromate 63x/1.4 Oil or Plan-Apochromate 100x/1.4 Oil objective as described previously [82, 83]. All images were processed using imageJ [84] and Cell profiler [85].

### Metabolomics sample preparation and analysis

Both extracellular and intracellular metabolites were analyzed in this study. For extracellular metabolite measurements, 20 μL of spent medium was dried *in vacuo* and kept in −80 °C before chemical derivatization. For intracellular metabolite measurements, frozen cells on membranes (see above) were transferred to a fresh microcentrifuge tube and 100 μL of nanopure water was added prior to vortexing. Metabolites were extracted with addition of 400 μL of chloroform/methanol mixture (2:1, v/v) and vigorous vortexing. After centrifugation (15,000 × g, 4 °C, 5 min), the aqueous layer was transferred to glass vials and dried in a vacuum concentrator. Chemical derivatization of extracted metabolites and subsequent GC-MS analysis were performed as reported previously [82, 86]. Briefly, dried metabolites were chemically modified with two derivatizations including methoxyamination and trimethylsilylation. The derivatized samples were analyzed by GC-MS, and the resulting data processed with MetaboliteDetector [87]. Retention times were calculated based on a mixture of fatty acid methyl esters.

### Protein isolation and enzymatic digestion

To increase the coverage of both the phosphoproteome and global proteome, two methods of protein isolation were performed – a standard protocol based on solubilization of proteins in buffer containing 8 M urea (protocol 1), and a protocol based on precipitation of protein using chloroform/methanol (as described above for metabolite extraction; protocol 2), followed by solubilization using the first protocol. Briefly, for protocol 1, a lysis buffer was added to each sample containing 8 M urea, 75 mM NaCL in 100 mM NH_4_HCO_3_ pH 7.8, and 10 mM NaF, 1 % cocktail 2 (Sigma, P 5726) and 1 % cocktail 3 (Sigma, P 0044) as phosphatase inhibitors. For protocol 2, a modified lysis buffer was added to the samples, which contained 1 % inhibitor cocktail 2 (Sigma, P 5726) and 1 % cocktail 3 (Sigma, P 0044) in 25 mM NH_4_HCO_3_. All samples were bead beat in a Bullet Blender (Next Advance, Averill Park, NY) at speed 8 for 3 min at 4 °C, and the lysate was spun into a falcon tube at 2000xg for 10 min at 4 °C. 0.5 mL of each sample was transferred into a fresh, Eppendorf tube and a BCA assay (Thermo Scientific, Rockford, IL) was performed to determine the protein concentration. A chloroform/methanol extraction was performed on six samples to precipitate protein. Briefly, −20 °C chloroform:methanol mix (prepared 2:1 (v/v)) was added to the samples in a 5:1 ratio over sample volume and vigorously vortexed. The samples were then placed on ice for 5 min and then vortexed for 10 s followed by centrifugation at 10,000xg for 10 min at 4 °C. The precipitated protein was removed and washed with 1 mL of ice cold methanol. The samples were centrifuged at 10,000xg for 10 min to pellet the protein and the methanol decanted off. The remaining protein was placed in a fume hood to dry. Lysis buffer (from protocol 1) was then added to the protein pellets. 10 mM DTT was added to all samples, followed by sonication and incubation at 60 °C for 30 min with constant shaking at 800 rpm. Samples were then diluted 8-fold for preparation for digestion with 100 mM NH_4_HCO_3_, 1 mM CaCl_2_, and sequencing-grade modified porcine trypsin (Promega, Madison, WI) was added to at a 1:50 (w/w) trypsin-to-protein ratio, followed by incubation for 3 h at 37 °C. Digested samples were desalted using a 4-probe positive pressure Gilson GX-274 ASPEC™ system (Gilson Inc., Middleton, WI) with Discovery C18 100 mg/1 mL solid phase extraction tubes (Supelco, St. Louis, MO), using the following protocol: 3 mL of methanol was added for conditioning followed by 2 mL of 0.1 % TFA in H_2_O. The samples were then loaded onto each column followed by 4 mL of 95:5: H_2_O:ACN, 0.1 % TFA. Samples were eluted with 1 mL 80:20 ACN:H_2_O, 0.1 % TFA. The samples were concentrated down to ~30 μL using a Speed Vac, and a final BCA was performed to determine the peptide concentration.

### Isobaric chemical labeling of peptides

The samples were measured and vialed to contain 100 μg each, and the volumes were brought up using 0.5 M TEAB (or dried down to) 15 μl in a low-protein binding 1.5 mL centrifuge tube. The pH of each sample was measured and brought to over pH 8 using 1 M TEAB. Vials of 4-plex iTRAQ reagent (AB Sciex, Framingham, MA) were brought to room temperature. The reagents were pulse spun to ensure the contents were collected at the bottom and 60 μl of isoproponal was added to each reagent vial. The reagents were thoroughly vortexed, spun down and added to the appropriate sample. Each sample was vortexed and spun down to incubate at room temperature for 2 h at which time 100 μL of nanopure water was added to hydrolyze the sample, followed by incubation for an additional 30 min. The samples were partially dried down in a speed vac to remove the organic solvent and then pooled together to obtain three samples each containing all 4 of the iTRAQ labels and C18 cleaned again, as described above, followed by another BCA assay to determine the final peptide mass for HPLC fractionation.

### Fractionation of labeled peptides

All samples were diluted to a volume of 900 μL with 10 mM ammonium formate buffer (pH 10.0), and resolved on a reversed-phase HPLC column (XBridge C18, 250x4.6 mm, 5 μM; Waters, Milford, MA) with a guard column (4.6x20 mm) of the same material (Waters). Samples were fractionated at 0.5 mL/min using an Agilent 1100 series HPLC system (Agilent Technologies, Santa Clara, CA) with mobile phases (A) 10 mM Ammonium Formate, pH 10.0 and (B) 10 mM Ammonium Formate, pH 10.0/acetonitrile (10:90, v/v). The gradient was adjusted from at 100 % A to 95 % A over the first 10 min, 95 % A to 65 % A over min 10 to 70, 65 % A to 30 % A over min 70 to 85, maintained at 30 % A over min 85 to 95, re-equilibrated with 100 % A over min 95 to 105, and held at 100 % A until min 120. Fractions were collected every 1.25 min (96 fractions over the entire gradient) in a 96-well plate. The plate was partially dried in a speed vac and then every 24th fraction was combined for a total of 24 samples (each with *n* = 4 fractions pooled), using 50 % acetonitrile in water to transfer fractions. The fractions were then completely *in vacuo* and 25 μL of 25 mM ammonium bicarbonate was added to each. The samples were then divided in half, and one half was placed into autosampler vials and stored at at −20 °C until LC-MS/MS analysis. The remaining half were further pooled into 4 fractions for phosphopeptide enrichment using immobilized metal-ion (Fe^3+^) affinity chromatography [88], as previously described [89, 90].

### LC-MS/MS analysis of peptides

A Waters Nano Acquity LC (Waters, Milford, MA) LC system was coupled to a LTQ Orbitrap Velos mass spectrometer (Thermo Scientific, San Jose, CA) and used for the proteomics analyses. Orbitrap spectra (AGC 1 × 10^6^) were collected from 300 to 1800 *m/z* at a resolution of 60 k HMS, profile mode, followed by data dependent Orbitrap HCD top 10 MS/MS, centroid mode, at a resolution of 7500. Tandem mass spectra were acquired with a normalized collision energy set to 45. The heated capillary temperature and spray voltage were 325 °C and 2.2 kV, respectively. Charge state screening was enabled to reject singly charged ions and the default charge state was set to five. The mass spectrometer was outfitted with a custom electrospray ionization (ESI) interface, using a custom made, chemically etched electrospray emitter,as previously described [91].

Data were acquired for 100 min after a 25 min delay from when the LC gradient started. Residual salts were first removed using a SPE column prepared in-house by slurry packing 3.6-μm Aeris Widepore C_18_ particles (Phenomenex, Torrance, CA) into a 5 cm × 360 μm o.d. × 150 μm i.d fused silica capillary (Polymicro Technologies Inc., Phoenix, AZ) using a 1-cm sol–gel frit on each end for media retention [92]. The reversed-phase column was prepared in-house by slurry packing 3.0-μm Jupiter C_18_ particles (Phenomenex, Torrance, CA) into a 70-cm × 360 μm o.d. × 75 μm i.d fused silica capillary (Polymicro Technologies Inc., Phoenix, AZ) using a 1-cm sol–gel frit for media retention. Mobile phases consisted of 0.1 % formic acid in water (A) and 0.1 % formic acid in acetonitrile (B) operated at a flow rate of 300 nL/min. The 5 μL injection volume was focused onto the analytical column and then eluted with a gradient profile as follows (min:%B); 0:0.1, 2:8, 20:12, 75:30, 97:45, 100:95 and held for 10 min before going back to starting conditions. The column was equilibrated for 52 min before the next sample was loaded onto the column.

### Proteomics data processing

Raw tandem mass spectra from the mass spectrometer were processed using DtaRefinery [93] coupled to MSGFPlus [94] and searched against a collection of *Y. lipolytica* proteins obtained from Uniprot. Briefly, a parent mass tolerance of +/− 20 ppm, dynamically phosphorylated (+79.966331 Da) serine, threonine or tyrosine, static iTRAQ 4-plex (+144.102066 Da) modification of lysine and peptide N-terminus, with partial tryptic cleavages were considered. An in-house analysis pipeline was used to combine the peptide identifications with their related iTRAQ 4-plex reporter ion abundances derived from MASIC [95]. Briefly, the pipeline imports the peptide identification results, filters the peptide results to 1 % FDR (using MSGFPlus’ reported Q-value < = 0.01, which is derived using the standard decoy approach [96]), sums reporter ion intensities per peptide across multiple strong cation exchange fraction within a given sample, and outputs the peptide sequence with associated reporter ion intensities for each fractionated samples. Proteins associated with each peptide are reported separately. For this dataset the occurrence of peptide sequences occurring in more than one protein were rare, but in those cases where redundancy did occur all proteins associated with a peptide were reported. For quantitative comparison purposes, only the first protein reference associated with a peptide was used for further protein level analyses.

### Bioinformatic analysis

ITRAQ reporter ion intensities for peptides were summed per protein. Each replicate was then mean centered and log_2_ transformed using Inferno [97], the results of which are presented in Additional file 1. Positive fold-change values represent metabolites and proteins up-regulated in C/N = 150 condition when compared with C/N = 10. Phosphorylation levels were calculated for individual phosphorylated peptides and normalized to their protein abundance level. Thus changes in phosphorylation state represent the fraction of the protein present that is phosphorylated. *Y. lipolytica* gene models were annotated using Blast2GO [98] and orthologs of *Y. lipolytica* genes were identified in *Saccharomyces cerevisiae* and *Neurospora crassa* by reciprocal BlastP analysis. For Blast2GO analysis, proteins changing in concentration with *p* < 0.001 following a two tailed *t*-test were considered as being up- or down-regulated during nitrogen limitation. For simplicity, genes were assigned the symbol of their ortholog in *S. cerevisiae* from SGD [99] when appropriate. Up- and down-regulated proteins were tested for enrichment of gene ontology terms using Fisher’s exact test. Fold-change quantities were mapped to the yli organism KEGG maps [100] to investigate trends identified by Blast2GO. Transcription start sites (TSS) were annotated by mapping high-throughput RNA-seq data, kindly provided by Jens Nielsen’s group prior to publication (Kerkhoven E et al., in prep), to the *Y. lipolytica* reference genome [25] using Bowtie [101] and Samtools [102]. The TSS for each gene model is defined as the point 5’ of the start codon when the read count diminishes to the background level. Promoter regions were defined as the 500 bp upstream of the TSS for identification of enriched motifs using DREME [103] and as the region upstream of the transcription start site to the next transcript for motif location analysis using custom Perl scripts. TOMTOM [104] was used to identify proteins potentially binding the enriched motifs.

### Availability of supporting data

All supporting data are included as additional supplementary files.

## Additional files

Additional file 1:
**Supplementary Figures.** Includes supplemental Figure S1, principal component analysis of intracellular and extracellular metabolite datasets; supplemental Figure S2, mean centered proteome quantification; supplemental Figure S3, global protein abundance level of phosphorylated and non-phosphorylated peptides identified after metal affinity chromatography. (PDF 334 kb) Additional file 2:
**Metabolite, global peptide, phosphopeptide, global proteome, phosphoproteome, and phosphosite quantification.** Tables contain dry-weight normalized metabolite relative quantities, mean-centered and log2 transformed global peptide quantities with and without methanol/chloroform utilization for extraction, mean-centered and log2 transformed phosphopeptide quantities with and without methanol/chloroform utilization for extraction, mean-centered and log2 transformed global protein quantities with and without methanol/chloroform utilization for extraction, mean-centered and log2 transformed phosphoproteins quantities with and without methanol/chloroform utilization for extraction, and global protein abundance normalized log2 fold change quantities for phosphopeptides. (XLSX 10435 kb)

## Abbreviations

bHLH: basic helix-loop-helix
GO: gene ontology
IMAC: immobilized metal affinity chromatography
TCA: tricarboxylic acid
TSS: transcription start site
