# Supplementary material for: Multi-omics analysis reveals regulators of the response to nitrogen limitation in Yarrowia lipolytica
Source: BMC Genomics. 2016 Feb 25;17:138. doi: 10.1186/s12864-016-2471-2 (PMC4766638; doi:10.1186/s12864-016-2471-2)
Supplement: Additional file 1: — Supplementary Figures. Includes supplemental Figure S1, principal component analysis of intracellular and extracellular metabolite datasets; supplemental Figure S2, mean centered proteome quantification; supplemental Figure S3, global protein abundance level of phosphorylated and non-phosphorylated peptides identified after metal affinity chromatography. (PDF 334 kb) [file 12864_2016_2471_MOESM1_ESM.pdf]

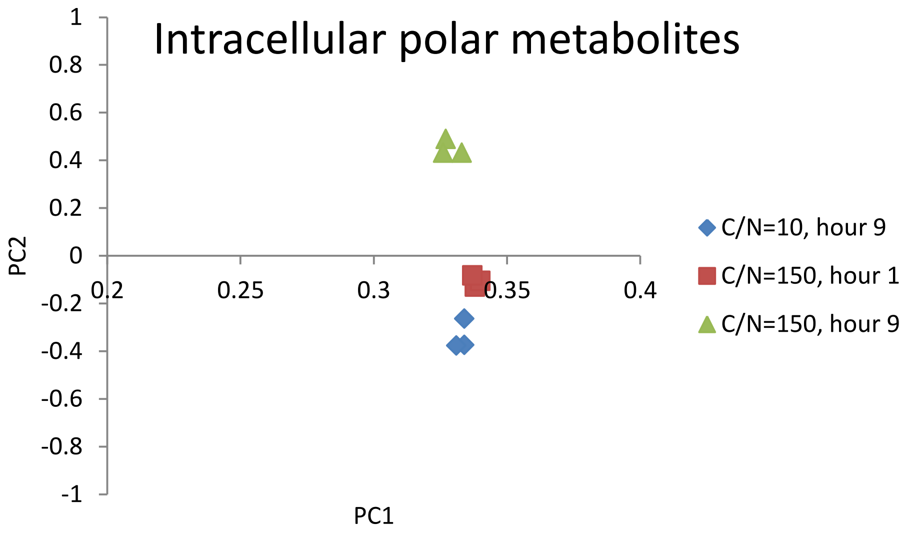

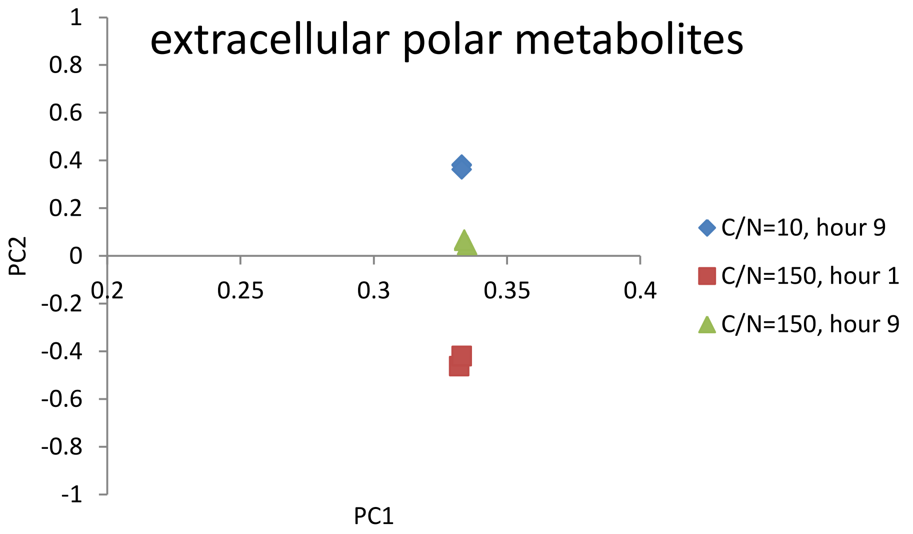

Supplemental figure 1. Principal component analysis of intracellular and extracellular metabolite datasets. Biological replicates are color coded and plotted along principal components 1 and 2 to visualize clustering and reproducibility.

A

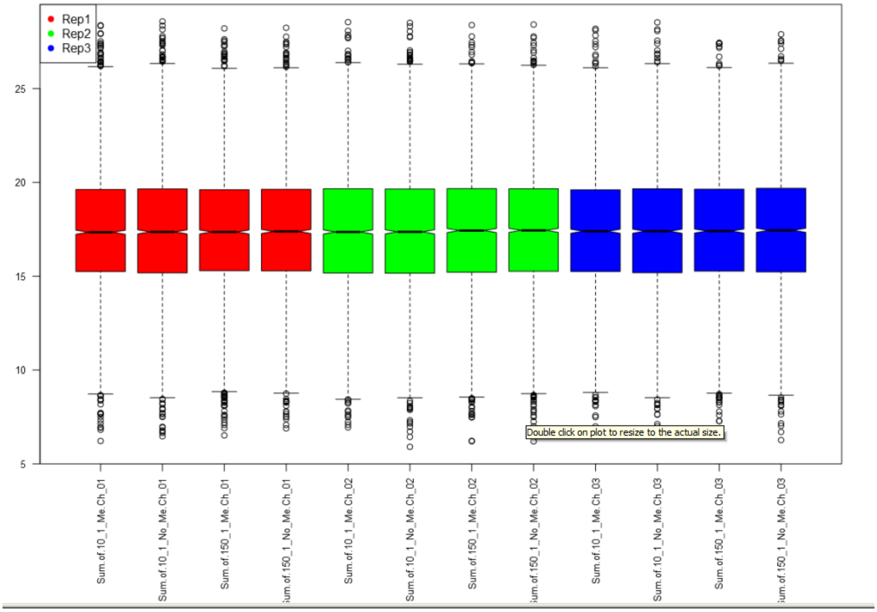

B

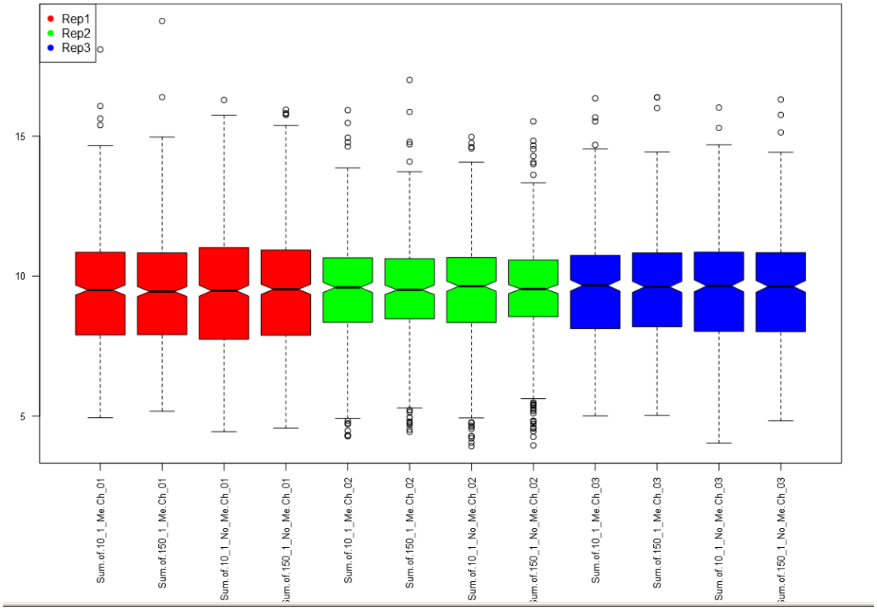

Supplemental figure 2. Mean centered proteome quantification. Raw data was log base 2 transformed and mean centered. Twelve samples (2 C/N conditions x 2 extraction methods x 3 replicates) were analyzed for each dataset and are colored according to replicate. A) Global proteome quantification. B) Phosphoproteome quantification.

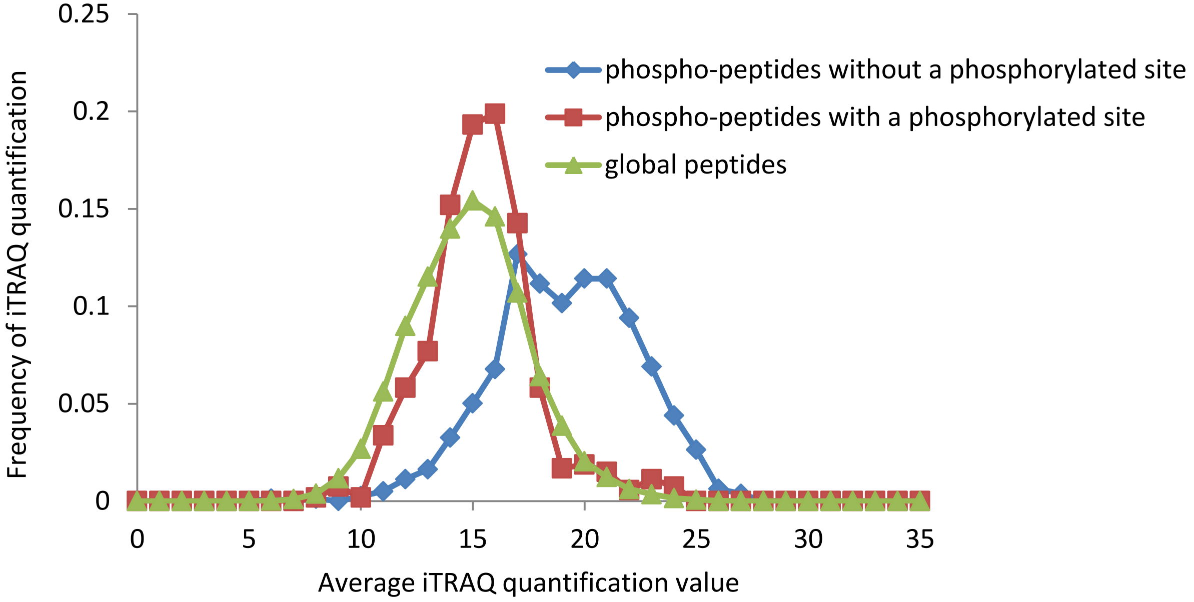

Supplemental figure 3. Global protein abundance level of phosphorylated and non-phosphorylated peptides identified after metal affinity chromatography. Non-phosphorylated peptides identified after metal affinity chromatography enrichment for phosphorylation are skewed toward the very high end of protein expression whereas the phosphorylated peptides identified are not when compared with all global peptides, suggesting the non-phosphorylated peptides are contamination due to their high intracellular concentration.
